# Local recurrences after curettage and cementing in long bone giant cell tumor

**DOI:** 10.4103/0019-5413.77138

**Published:** 2011

**Authors:** Kabul C Saikia, Tulsi D Bhattacharyya, Sanjeev K Bhuyan, Bikas Bordoloi, Bharat Durgia, Firoz Ahmed

**Affiliations:** Department of Orthopaedics, Gauhati Medical College, Guwahati, Assam, India

**Keywords:** Bone cementing, GCT, recurrence

## Abstract

**Background:**

Giant cell tumor of bone (GCT) is a benign lesion with great propensity for local recurrence. This study aimed to analyse the rates of local recurrence and its possible predisposing factors in Campanacci’s Grade III and II GCT of long bones following intralesional curettage and bone cementing.

**Materials and Methods:**

32 cases of either sex with Campanacci’s Grade II (n= 14), and Grade III (n=18) with intact articular surface, operated between 1995 and 2007 in form of intralesional curettage and bone cementing were studied. All the cases were followed up for 2.5-12 years (mean, 6.5), after primary treatment. The mean age at operation was 32.4 years (range, 18.5-40 years). The proximal tibia was involved in 13 cases (40.6%), followed by distal femur (n=11)34.4% distal tibia (n=3) 9.4%, proximal femur (n=2) 3.2% and distal radius (n=3) 9.4%.

**Results:**

Eleven patients (34.4%) had local recurrence, of which eight were of Campanacci’s Grade III. The mean recurrence time was 14 months (range, 3-34 months). The two-year recurrence-free survivorship was 71.9% (n=23/32). Post-recurrence mean follow-up was 4.2 years (range, 2-6.5 years).

**Conclusion:**

We observed higher rate of local recurrence with Campanacci’s Grade III GCTs. We recommend selective use of this procedure in Grade III lesions, particularly with extensive soft tissue involvement.

## INTRODUCTION

Giant cell tumour of the bone (GCT) is a benign but locally aggressive neoplasm with a tendency of local recurrence.^1^ The method of curettage with polymethylmethacrylate (PMMA) cementing, which was first described in 1969,^2^ has gained wide acceptance for the treatment of large juxta-articular GCTs. The polymerisation of PMMA produces a local chemical cytotoxic effect.^3^ Progressive lysis or absence of sclerotic rim at bone-cement interface may suggest recurrence.^4^ Detection of recurrence is easier as lysis always occurs on the extra-lesional site of the bone-cement interface.^5^,^6^

The prevalence of local recurrence in large single centre studies in GCTs with curettage and bone cementing are in the range 0-29%, with a minimum follow-up of two years.^7^–^10^ The low recurrence rate is not only due to tumoricidal effect of the cement but also because of the adequacy of tumor removal.^11^ Current recurrence rate of 10-20% following meticulous curettage and extended tumor removal using mechanised burr and adjuvant therapy is a vast improvement.^12^– ^14^

The objective of this retrospective study is to analyse any difference in local recurrence rate and document the factors that might predispose to recurrence in Grade III and Grade II lesion of long bones primarily treated with intralesional curettage and bone cementing.

## MATERIALS AND METHODS

Between 1995 and 2007, we treated consecutive 37 patients of Campanacci’s Grade III and Grade II GCT, with a mean follow-up period of 6.5 years (range, 2.5-12 yrs). We evaluate these cases retrospectively.

Campanacci grading was used; Grade I as well-defined tumor with radio-opaque rim, Grade II as well-defined margins with moderately expanded but intact cortex and no radio-opaque rim and Grade III as ill-defined margins with soft tissue mass.^15^ The primary modality of treatment was intralesional curettage and bone cementing. No other adjuvants were used. Inclusion criteria were: Patients with Grade II and Grade III lesions that did not invade the joint who had undergone primary treatment at our institute and a minimum of two years followup was available, less than 50% of the cortex was destroyed with any extraosseous mass or destruction of cortex. Plain radiographs, chest X-ray, computed tomography (CT) and/or magnetic resonance imaging (MRI) in more than one plane. Fine needle aspiration cytology (FNAC) and/or Open biopsy, were done in all cases. Thickness of the subchondral bone at adjacent articular surface was measured radiologically.

Exclusion criteria were: Patients with Campanacci’s Grade III tumor with joint involvement, breaking through the cortex in two planes or more than 50% of the surrounding metaphysis destroyed. Five patients were lost to follow-up within two years, leaving 32 patients for evaluation. There were 18 men and 14 women, with mean age at operation being 32.4 years (range, 18-54 years).Two of our Grade III patients presented with pathological fracture (Case 14,19). Patient details are given in Table 1.

**Table 1:** Clinical details of patients

Age/sex (yrs)	Site	Grade of tumor (Campanacci’s)	Recurrence	Re-recurrence
22/M	D/F	III	-	-
18/F	P/T	III	-	-
31/M	D/F	III	+	-
27/F	D/F	II	-	-
40/M	P/T	III	-	-
32/M	P/F	II	-	-
38/F	D/F	III	+	+
27/F	P/T	III	+	-
39/M	D/F	II	-	-
37/M	D/R	II	+	-
29/F	P/T	III	-	-
32/F	D/T	II	-	-
35/M	P/T	III	+	-
30/M	D/F	III	-	-
36/M	D/R	II	-	-
32/F	P/T	III	+	-
28/M	P/T	III	-	-
24/M	P/F	II	+	-
32/F	D/F	III	-	-
38/M	P/T	II	-	-
36/F	D/T	III	+	-
39/F	D/F	II	-	-
27/M	P/T	III	-	-
35/F	P/T	III	-	-
39/F	P/T	II	+	+
36/F	D/F	III	-	-
28/M	D/R	II	-	-
31/M	P/F	III	+	-
37/F	P/T	II	-	-
29/M	D/F	II	-	-
34/M	P/T	II	-	-
39/M	D/F	III	+	-

D/F: Distal femur; P/F: Proximal femur; P/T: Proximal tibia; D/T: Distal tibia; D/R: Distal radius; F: Female; M: Male; yrs: years

CT was done in tumors which showed no cortical break in X-ray, while MRI was done in patients showing cortical break in either X-ray or the subsequent CT.

**Figure 1:**
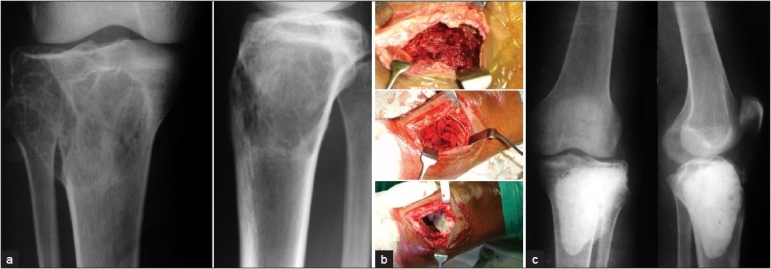
(a) X-ray (anteroposterior and lateral views) showing GCT of upper end of tibia, (b) intra-op photo showing (from above downwards) cavity after curettage, cancellous bone grafting, gel foam packing before cement application. (c) Post-operative X-ray (anteroposterior and lateral views) showing bone cement *in situ*

Incision was made near the site of cortical break or near the area with maximum cortical thinness. The tumor tissue was scooped out through a large window with the help of hand-held curettes. The lesion walls were than treated with high-speed burr to break the bony ridges. The curettage was considered complete after normal cortical bone and medullary cavity was visible. The curetted material was sent for routine histopathological examination. A pulsatile jet lavage was used at the end to wash out tumor cells and the cavity was then manually packed with standard PMMA. No chemical adjuvant was used. Cooled saline solution was used to irrigate the area and joint surface to prevent thermal damage to the articular surface. Special care was taken in Grade III tumors with soft tissue involvement and the tumor along with the soft tissue mass till healthy soft tissue was visualised and axcised. Lesions around weight bearing joints with less than five mm of subchondral bony support (n=5) were reinforced with autogenous iliac graft of two to three mm thickness Sandwich technique). A layer of gel foam was placed over the graft and the remaining cavity was packed manually with bone cement [Figure 1]. No implant was used in the study. The cases with pathological fracture were treated with curettage and cementing and the cement was thought to provide adequate stabilisation. However, weight bearing in these cases was delayed by up to 4–6 weeks. Early mobilisation and weight bearing usually after 1 week^16^ was encouraged in all patients after the pain subsided. The cases were followed up at six-week intervals until six months, then at three-month intervals till one year and then at six-month intervals with X-rays and CT scans.

Recurrence was diagnosed when there was progressive lysis of more than 5 mm at the bone-cement interface or if sclerotic rim at the bone-cement interface was absent.^16^,^17^

Possible factors that might influence the recurrence, such as age and sex, location of the tumor, presence of pathological fracture and Campanacci’s grading were recorded. Also, radiographic deterioration of articular cartilage was analysed as a possible complication of cementing. Radiographic deterioration of articular cartilage was assessed by marginal osteophytes, joint space narrowing, subchondral sclerosis, and subchondral cysts on X-ray.

## RESULTS

We had 18 cases were of Campanacci’s Grade III and 14 of Grade II. A majority of Grade III tumors (n=10; 55.6%) had cortical breakthrough with massive soft tissue involvement. Out of 14 cases of Grade II lesions, 4 were in distal femur and proximal tibia each. A majority of the Grade III lesions (n=9; 50%) were in proximal tibia. The mean operating time was 1.4 hrs (range, 1.2-2 hrs.).

**Figure 2:**
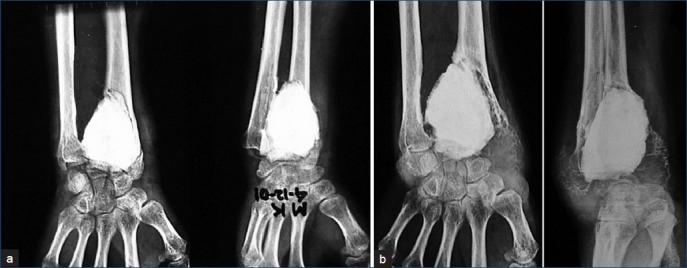
X-rays (anteroposterior and oblique views) of wrist joint showing (a) Two-weeks postoperative X-ray showing bone cementing after curettage. (b) Recurrence after bone cementing at 12 months

Eleven patients had local recurrence (34.4%). The mean interval between surgery and local recurrence was 14 months (range, 3-34 months) with 6 reported with recurrence after one year [Table 2]. The highest rate of local recurrence was in distal radius (n=2/3; 66.6%), followed by distal tibia (n=1/2; 50%), distal femur (n=5/11; 45.5%) and in proximal tibia (n=3/13; 23.07%) [Figure 2]. No recurrence was detected in proximal femoral GCTs (2 grade II and 1 grade III). Of 11 recurrences, Grade III lesions accounted for eight cases (n=8/18; 44.4%) (p value=0.966) and Grade II for three cases (21.4%). Among the eight cases of Grade III lesions, five presented with cortical breakthrough with massive soft tissue involvement. There was no malignant recurrence. Patients with recurrence were treated with secondary procedure like intralesional curettage and re-cementing (n=8/11; 72.7%) and wide resection (n=3/11; 27.3%). The rate of recurrence was independent of age and sex of the patient. The two-year recurrence-free survivorship was 71.8% (n=23/32), with Grade II 85.7% (12/14) and Grade III 61.1% (11/18). Post-recurrence follow-up was 2-6.5 years (mean, 4.2 years).

**Table 2:** Clinical details of patients with recurrence

Case no.	Time of recurrence (months)	Treatment of recurrence	Re-recurrence (months)	Treatment of recurrence
1.	05	BC	-	-
2.	08	BC	9	BC
3.	18	BC	-	-
4.	12	BC	-	-
5.	03	Wide resection	-	-
6.	16	BC	-	-
7.	28	BC	-	-
8.	10	Wide resection	-	-
9.	06	BC	16	Wide resection
10.	34	Wide resection	-	-
11.	14	BC	-	-

BC: Bone cementing

Re-recurrence was observed following secondary procedure in two cases (n=2/11; 18.1%). Both re-recurrences were following secondary procedure of cementing (n=2/8; 25%). The re-recurrence was detected at 9 and 16 months, respectively after second recurrettage and cementing. One was treated with curettage and cementing while the other underwent a wide resection procedure. None of them has shown any further evidence of recurrence till the most recent follow-up. Seven of the 11 recurrence cases gave consent for the inspection of the articular cartilage arhtroscopically and no evidence of pathological changes was detected.

## DISCUSSION

Limited information is available about the risks of recurrence following curettage and bone cementing in Grade II and III GCTs of the long bone.^18^ Most of the recurrences (80-97%) following primary treatment reported to occur within two years.^15^,^20^–^22^ Curettage has been advocated in GCT up to Grade III tumors where there is no joint invasion, less than 50% metaphyseal destruction and soft tissue mass in one plane only.^18^ Extended curettage was advocated when atleast 2 mm of subarticular bone was free of the tumor with no soft tissue spillage as assessed on a recent MRI^23^.

Exothermic reaction of PMMA generates local hyperthermia, which induces necrosis of any remaining neoplastic tissue without causing any local complication.^3^,^24^ Curettage and packing with bone cement has advantage to its association with low rate of recurrence and it provides immediate support and allows for intensive curettage even in the case of large tumor cavities.^25^ The additional advantages are low cost, ease of use, lack of donor-site morbidity, elimination of the risk of transmission of disease associated with allograft. It facilitates the radiographic detection of local recurrence earlier and easier. Adequate removal of the tumor seems to be more an important predictive factor for the successful outcome of primary surgery. Thus, use of high-speed burr is helpful and encouraging.^14^

Some of the small series reported to have no initial local recurrence.^15^,^26^,^27^ Conrad *et al*. reported five recurrences in 17 cases following curettege and bone cementing.^9^ In a multicentre study of 187 patients, Capanna *et al*. reported 17% recurrence rate^13^ while Remedios *et al*., reported four recurrences in 13 operations on 11 patients.^4^ Takuro Wada **et al**. reported recurrence in one case 24 months postoperatively from 15 patients treated with cementing.^28^ Wang **et al**. had a series of 14 cases of Grade III GCT with intralesional curettage.^29^ Four of them had bone cementing with local recurrence reported in one case at 21^st^ month of follow-up. O’Donell **et al**. reported 25% recurrence rate (n=15/60) following curettage and packing with PMMA cement.^30^ The local recurrence in their study was found to be 23% in Grade II tumors (n=9/40) and 36% in Grade III tumors (n=6/16). Our study shows much higher recurrence rate in Grade III tumor (44%) compared to Grade II tumors (21.4%). Local recurrence reported by O’Donell **et al**. using bone cement and curettage was 33.3%, which decreased to 16.6% when mechanical burr was used. We used high-speed burr routinely in all cases. Using curettage with high-speed burring and cementing, recurrence rate was variable with different authors. Jamshidi **et al**. reported 16.7% (n=7/42) recurrence rate and Lim **et al**. found it to be 44% (n=4/9).^31^,^32^

Predisposing factors for recurrence are short duration of symptoms less than 2 months, early radiographic cortical destruction with minimal lesion, marked soft tissue swelling and location of the tumor. Age, sex and pathological fracture cannot be correlated with recurrence.

Our study shows highest rate of local recurrence in the distal radius (n=2/3; 66.7%) followed by distal tibia (n=1/2; 50%), distal femur (n=5/11; 45.5%) and proximal tibia (n=3/13; 23%), but the results are not statistically significant because of less number of patients in each subgroup. O’Donell also found the highest local recurrence in distal radius (n=5/10; 50%).

Although early degeneration of cartilage following bone cementing may occur,^30^ such degeneration has not been observed in our study. Routine iliac crest bone grafting was done in patients with less than 5 mm of subchondral bone with the aim of preventing early degenerative changes. Our cases of pathological fractures (n=2) did not have any recurrence. Pathological fractures through GCTs are not a contraindication to treatment by curettage and cementing.^33^,^34^ No implants were used. The cases with pathological fracture were also treated with curettage and cementing, and the cement was thought to provide adequate stabilisation. However, weight bearing in these cases was delayed by up to 4–6 weeks.

**Figure 3:**
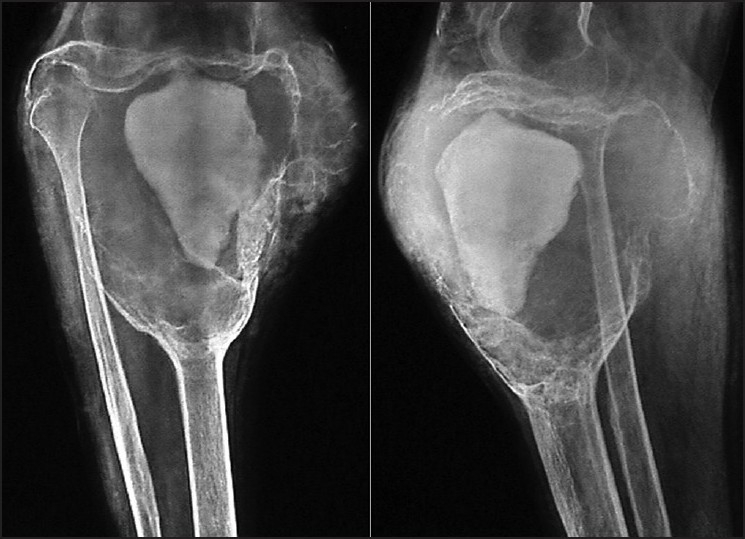
X-rays (anteroposterior, lateral views) of proximal both bones leg with knee joint showing recurrence at 28 months after initial refusal for surgery

Recurrent lesions are to be treated by the same principle as primary. Most of our local recurrence cases (n=8) were treated with bone cementing like primary tumors.^11^,^35^–^38^ However, Case 16 presented with recurrence at 16 months but refused surgery. Figure 3 shows her status at 28 months. Many authors favour repeated curettage for recurrent lesion and others prefer more extensive surgery.^20^,^39^ The Yu **et al**.,^38^ reported recurrence rate of the patients treated for recurrence with secondary curettage as 46% (n=6/13).Recurrence rate of patients referred for the treatment of GCT recurrence was much higher than that for primary cases in their report. Our study had 25% recurrence (n=2/8) rate in patients who were treated for recurrence with secondary curettage and cementing.

Our rate of local recurrence (34.47%) is higher than that reported by most authors.^7^–^10^ Various possibilities exist for increased percentage of recurrence in our study. A large number of Grade III tumors had cortical break through with massive soft tissue involvement (n=10), where the recurrence rate was found to be 50% (n=5/10). Minimal cortical break with extension/massive tissue involvement indicates more aggressiveness.^40^ The study includes a cross-section of all patients who had undergone treatment for GCTs of long bone at our institute rather than being limited to selected cases who had Grade II or less aggressive tumors. This may also explain the much higher recurrence rate in our series. Vander G and Lachman advocated curettage and bone cementing in GCTs, with minimal cortical perforation with 0 and 6% recurrence rates, respectively.^27^,^40^ If the cortex is deficient radiologically, the curettage and bone cementing has higher recurrence. It may be because soft tissue infiltration has already taken place at the time of presentation.^41^ The influence of operative technique in the rate of recurrence is also important.^24^,^27^ Our duration of post-recurrence follow-up is 4.2 years with acceptable re-recurrence rate of 18.1%.

## CONCLUSION

This study reveals a higher rate of recurrence following intralesional curettage and bone cementing in Grade III GCTs. Therefore, we believe that one should be selective in the use of this procedure in Grade III lesions.
